# ESAT-6 Targeting to DEC205+ Antigen Presenting Cells Induces Specific-T Cell Responses against ESAT-6 and Reduces Pulmonary Infection with Virulent *Mycobacterium tuberculosis*

**DOI:** 10.1371/journal.pone.0124828

**Published:** 2015-04-27

**Authors:** Aarón Silva-Sánchez, Selene Meza-Pérez, Adriana Flores-Langarica, Luis Donis-Maturano, Iris Estrada-García, Juana Calderón-Amador, Rogelio Hernández-Pando, Juliana Idoyaga, Ralph M. Steinman, Leopoldo Flores-Romo

**Affiliations:** 1 Department of Cell Biology, Cinvestav-IPN, Ciudad de México, Mexico; 2 Department of Immunology, ENCB-IPN, Ciudad de México, Mexico; 3 Physiology and Cell Biology, Rockefeller University, New York, New York, United States of America; 4 Department of Pathology INNSZ, Ciudad de México, Mexico; Institut Pasteur, FRANCE

## Abstract

Airways infection with *Mycobacterium tuberculosis* (Mtb) is contained mostly by T cell responses, however, Mtb has developed evasion mechanisms which affect antigen presenting cell (APC) maturation/recruitment delaying the onset of Ag-specific T cell responses. Hypothetically, bypassing the natural infection routes by delivering antigens directly to APCs may overcome the pathogen’s naturally evolved evasion mechanisms, thus facilitating the induction of protective immune responses. We generated a murine monoclonal fusion antibody (α-DEC-ESAT) to deliver Early Secretory Antigen Target (ESAT)-6 directly to DEC205^+^ APCs and to assess its *in vivo* effects on protection associated responses (IFN-γ production, *in vivo* CTL killing, and pulmonary mycobacterial load). Treatment with α-DEC-ESAT alone induced ESAT-6-specific IFN-γ producing CD4^+^ T cells and prime-boost immunization prior to Mtb infection resulted in early influx (d14 post-infection) and increased IFN-γ^+^ production by specific T cells in the lungs, compared to scarce IFN-γ production in control mice. *In vivo* CTL killing was quantified in relevant tissues upon transferring target cells loaded with mycobacterial antigens. During infection, α-DEC-ESAT-treated mice showed increased target cell killing in the lungs, where histology revealed cellular infiltrate and considerably reduced bacterial burden. Targeting the mycobacterial antigen ESAT-6 to DEC205^+^ APCs before infection expands specific T cell clones responsible for early T cell responses (IFN-γ production and CTL activity) and substantially reduces lung bacterial burden. Delivering mycobacterial antigens directly to APCs provides a unique approach to study *in vivo* the role of APCs and specific T cell responses to assess their potential anti-mycobacterial functions.

## Introduction

*Mycobacterium tuberculosis* (Mtb), the causative agent of Pulmonary Tuberculosis (TB), is one of the oldest human pathogens known [1,2]. Among the glut of immune evasion mechanisms evolved in Mtb, the ability to subvert antigen presentation to CD4+ and CD8+ T cells, key mediators of Mtb immunity, is thought to be a critical barrier to developing a successful immunization strategy. Cytokine production by Mtb-specific CD4^+^ T cells helps control Mtb infection by activating and inducing NO production by macrophages [3–5] and by inducing Mtb-specific cytotoxic CD8 T cells [6,7]. In fact, IFN-γ production by T cells is necessary for containing pulmonary Mtb infection [8–11].

Mtb uniquely targets alveolar macrophages (AM) and lung dendritic cells (DC) to disrupt and delay antigen presentation to T cells in the draining lymph node (Mediastinal LN). DCs and AMs, both constituting the majority of lung antigen presenting cells (APC), defend against pulmonary infection by phagocytosing foreign particles and presenting these antigens to immune cells. Mtb specifically disrupts the function of lung APCs by causing the arrest of phagosome maturation [12,13], inhibition of phagosome-lysosome fusion [14,15], inhibition of cytotoxicity [16,17], and subversion of MHC-II intracellular trafficking[18]. Furthermore, Mtb delays the maturation and migration of lung dendritic cells [19–22]. Ultimately this results in delayed Mtb-specific T cell responses (17–20). In the experimental murine tuberculosis model, strong T cells responses are generated after 21 days of infection, the bacilli are not completely eliminated from the host and sterilizing immunity is not achieved.

However, evidence from murine tuberculosis models, suggest that accelerating the onset of IFN-γ producing-T cell responses can aid in control of Mtb[23]. For instance, increased T cell responses and reduced lung bacterial burden are achieved in mice immunized with recombinant mycobacterial proteins[24], infected with reconstituted attenuated bacteria[25], or after passive transfer of Mtb-specific T cells[5]. Given the disruption in antigen processing and presentation caused by Mtb, we have the hypothesis that targeting Mtb antigens to lung APCs would accelerate Mtb-specific T cell responses and hamper Mtb growth. Antigen targeting using monoclonal antibodies directed to DCs and coupled with a selected antigen is an effective way to induce strong, specific T cell responses [26,27]. In the case of pulmonary tuberculosis, lung DCs expressing DEC205^+^ are a potential candidate to deliver mycobacterial antigens since it has been shown *in situ* that DEC205^+^ DCs interact with virulent Mtb H37Rv bacilli, both in the lungs and in the mediastinal lymph nodes during airways infection [28]. Additionally, DEC205 is an endocytic receptor[29–31] associated with Ag processing and presentation[32,33], Mtb recognition[34], and, quite pertinent for this intracellular infection, with the induction of Th1-type CD8^+^ responses too [35].

In the present work we generated a murine monoclonal fusion antibody containing the mycobacterial antigen ESAT-6 and the APC targeting antibody, anti-DEC205, and evaluated its ability to speed Mtb-specific T cell responses and protection. Ligation of DEC205 by anti-DEC205-containing fusion mAbs induces endocytosis of the fusion mAb and subsequent TAP-dependent presentation of the Ag contained on the fusion mAb (31–33, 29). We chose to include the Mtb protein ESAT-6 as the antigen in our fusion mAb because it is a highly immunogenic mycobacterial antigen[36,37], that has been associated with strain virulence [38], induction of Th1 T cell responses, and contains a conserved, well-defined T cell epitope[39–41]. For instance, immunization with ESAT-6 alone or with ESAT-6-reconstituted BCG has shown *in vivo* protection to subsequent mycobacterial infection[42,43].

In our work, we targeted ESAT-6 to DEC205^+^ APCs (α-DEC-ESAT) and tested the *in vivo* effects upon cellular immune responses (IFN-γ production, *in vivo* CTL killing rate and lung Mtb clearance) during experimental pulmonary tuberculosis. To evaluate the ability of our fusion mAb, α-DEC-ESAT, to accelerate Mtb-specific T cell responses and contain Mtb, we prime-boosted with α-DEC-ESAT and poly-IC before airways infection, and evaluated Mtb-specific IFN-γ T cell responses, *in vivo* CTL killing, cellular infiltrate by lung histology, and bacterial burden in α-DEC-ESAT-treated mice and controls. We found that ESAT-6 targeting to DEC205^+^ APCs sped the onset and increased the magnitude of specific Th1-type T cell responses (IFN-γ production and *in vivo* CTL killing), and improved lymphocyte recruitment to the lungs of Mtb-infected mice. Furthermore α-DEC-ESAT treatment reduced pulmonary Mtb burden. These results indicate that direct antigen targeting to APCs can be an efficient strategy to increase and improve T cell responses during Mtb infection, and perhaps, to counteract Mtb naturally evolved evasion mechanisms.

## Materials and Methods

### Cloning and production of fusion α-DEC-ESAT mAb

**Fig 1:**
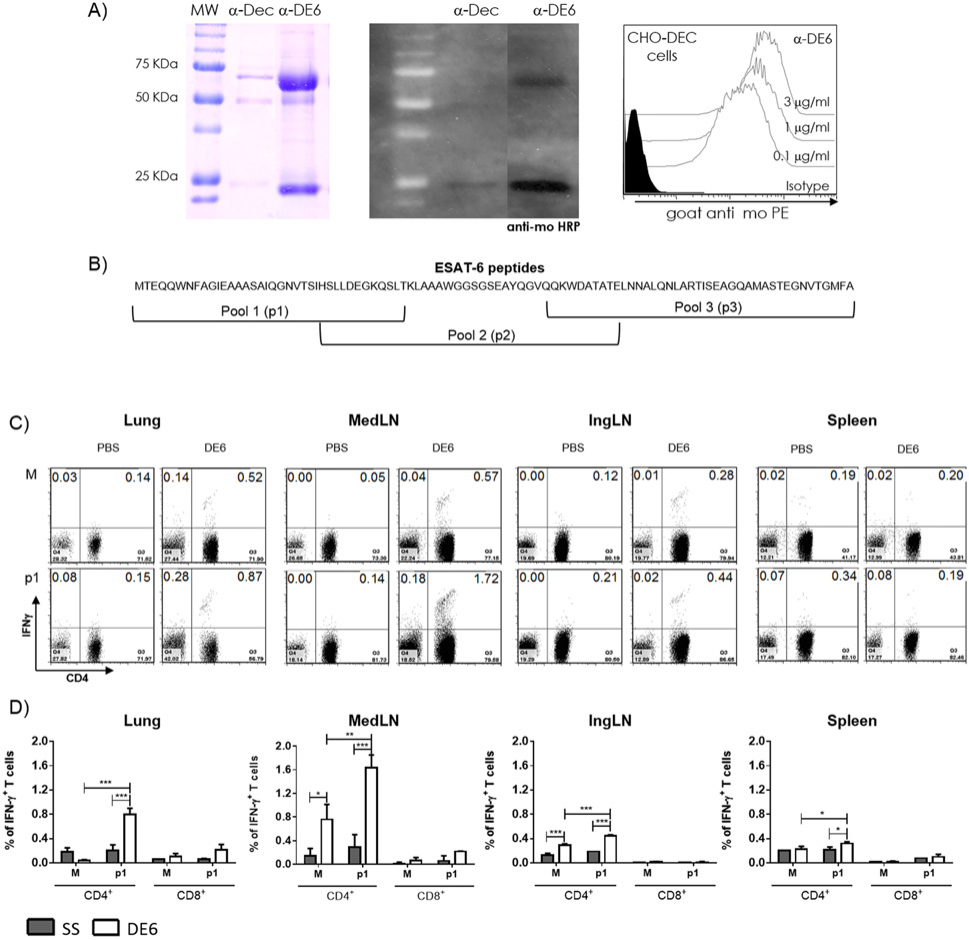
Immunization with α-DEC-ESAT hybrid antibody induces Th1 responses to ESAT-6 in the lungs and mediastinal lymph nodes of non-infected mice. A) A fusion antibody was generated to murine DEC205, genetically coupled to ESAT-6 and produced by transfected 293T cells. This Ab (α-DE6) presented an electrophoretic delay (left and middle figures), while binding to surface DEC205 on DEC205-transfected CHO cells was not affected (FACS histograms in the figure to the right). B) The ESAT-6 peptide library is depicted here to illustrate the distribution of the three pools of peptides that were used to stimulate cell suspensions from the different tissues examined, as indicated. Control unstimulated cell suspensions treated with culture medium alone are indicated with (M) while those treated with ESAT-6 pool 1 of peptides are indicated with (p1). C) and D) The IFN-γ production by ESAT-6-specific CD4^+^ T cells is shown as dot plots in (C), and as integrated results of the experiments performed for the various tissues assessed in (D). Data are presented as mean plus standard error and percentage of IFN-γ producing T cells. (*) represents P<0.05; (**) indicates P<0.01; (***) represents P<0.001. All bars represent uninfected mice treated with α-DEC-ESAT (white bars) or untreated (gray bars). MedLN = Mediastinal lymph nodes, IngLN = Inguinal lymph nodes.

Whole ESAT-6 sequence (obtained from *Mycobacterium bovis*) was cloned in frame into the COOH terminus of the monoclonal mouse anti—DEC205 and the isotype control mAb heavy chains. Fusion mAbs were produced by transient transfection (calcium phosphate) in 293T cells, purified on high-performance nickel sepharose columns (GE Healthcare), and fusion was confirmed by SDS—polyacrylamide gel electrophoresis (PAGE) (Fig 1A). The fusion antibodies were tested by flow cytometry for binding to CHO cells transfected to express the mouse DEC205 receptor (Fig 1A). Binding was revealed using a phycoerythrin conjugated goat α-mouse IgG secondary antibody (Biosource, Camarillo, CA).

### Experimental model of airways-induced pulmonary tuberculosis in mice

*M*. *tuberculosis* H37Rv was grown in Middlebrook 7H9 medium (Difco Laboratories) supplemented with OADC (Difco Laboratories). After 1 month of culture, mycobacteria were harvested, adjusted to 2.5x10^5^ bacteria in 100μl sterile endotoxin-free saline solution (SS), aliquoted, and maintained at -70°C until used. Before use, bacteria were recounted and their viability checked. We used the murine model of airways infection as described before, with some modifications[44]. Briefly, male BALB/c mice from 6–8 weeks of age were anaesthetized with sevoflurane (Abbott laboratories), then 2.5 x 10^5^ viable bacilli were inoculated in 100 μl sterile SS using an intra-tracheal probe to ensure delivery into the airways. Control animals were treated exactly the same except that were inoculated only with sterile SS. All animal work were performed in accordance to the guidelines of the Mexican constitution law NOM 062-200-1999, and approval of the Ethical Committee for Experimentation in Animals of the National Institute of Medical Sciences and Nutrition in Mexico (CINVA), permit number: 224. Mice were then maintained in cages fitted with microisolators in a P-3 biosecurity level facility.

Male BALB/c mice, 6–8 weeks old, were purchased from Jackson Laboratories. Priming immunization was done by footpad injection of 5μg of α-DEC-ESAT mAb and 10μg of poly I:C as adjuvant. Four weeks later intranasal boost immunization was given using the same dose of fusion antibody and adjuvant. Two weeks after boosting, mice were challenged with virulent Mtb H37Rv as described above. Control groups of mice received similar doses of poly I:C combined with isotype control mAb attached to ESAT (Iso-ESAT), or PBS, under the same immunization protocol.

### Detection of ESAT-6-specific IFN-γ producing T cells by flow cytometry

Spleen, lungs, mediastinal and inguinal lymph nodes cell suspensions were stimulated with one of three different ESAT-6 pool peptides (2μg/μL) in the presence of 1μg/μl costimulatory α-CD28 (clone 37.51) for 6h at 37°C, adding Brefeldin A (BFA) (10 μg/ml) for the last 4h to allow accumulation of intracellular cytokines. Cells were washed, incubated 10 min at 4°C with Power Block reagent to block Fc receptors, washed, and stained with fluorochrome-coupled mAbs for 15 min at 4°C. Cells were fixed, permeabilized, and stained with an α-IFN-γ mAb for 15 min at room temperature in Permwash 1X, resuspended in FACS buffer and 100,000 live CD3^+^-gated cells were acquired on a Dako Cyan Flow Cytometer. Data were analyzed with FlowJo Software (Tree Star, Inc., San Carlos, CA). As positive controls for IFN-γ production we used cell suspensions stimulated with α-CD3 and α-CD28 antibodies.

### Antibodies, reagents, and ESAT-6 peptide library

Monoclonal antibodies α-CD3-FITC (BD Pharmingen 553062), α-CD4-PerCP (BD Pharmingen 553052), and α-IFNγ-APC (BD Pharmingen 554413) were obtained from BD Biosciences (San Jose, California, USA). Intracellular staining was performed using the BD permeabilization kit Cytofix/Cytoperm—Permwash (BD Biosciences 554714). CFSE (21888) and PKH26 (PKH26-GL) fluorochrome labels were purchased from Sigma-Aldrich (Saint Louis, MO, USA) and cell staining was performed as suggested by manufacturers. BFA was obtained from Sigma-Aldrich (St. Louis, MO), and PowerBlock reagent from Biogenex (HK085-5K). ESAT-6 library of overlapping peptides (staggered by 4 amino acids spanning the entire ESAT-6) were synthesized by the Proteomics Resource Center (The Rockefeller University). The ESAT-6 peptide library was resuspended at 1 mg/ml of each peptide in 100% DMSO, the library was divided into three pools of 9–8 peptides each, spanning amino acids 1–36 (pool 1), amino acids 25–65 (pool 2), and amino acids 57–96 (pool 3) (Fig 1B).

### Quantification of mycobacterial Colony-Forming Units (cfu) in lungs

Lungs were homogenized using a polytron homogenizer (Kinematica, Luzern, Switzerland) then diluted with 0.05% tween-80 to a final volume of 1mL. Three consecutive logarithmic dilutions were made from this homogenate and 10μL of each dilution were plated by duplicate on Bacto Middlebrook 7H10 agar (Difco, Detroit, MI, USA) enriched with oleic acid, albumin, dextrose, and catalase. Plates were then incubated at 37°C and 5% of CO_2_ for 21 days. Total lung CFU was determined by adjusting colony counts, performed visually under dissecting microscope, to the dilution factor and final volume.

### Lung histology

For histological evaluations, the lungs were infused with 80% OCT in PBS, flash frozen in liquid nitrogen, and preserved at -80 C. 7μm cryosections were obtained in a Leica CM 1900 cryostat and stained with hematoxylin and eosin (Sigma-Aldrich). The area of lung tissue affected by cellular infiltrate was measured with Zidas Zeiss image analysis system. A minimum of two sections per lung per group was were used.

### *In vivo* target cell killing during airways infection with *M*. *tuberculosis* H37Rv

To trace the killing of target cells loaded with different mycobacterial antigens, we labelled splenocytes obtained from a naïve mouse using the combination of two fluorescent dyes (PKH26, red fluorescence; CFSE, green fluorescence). Two types of target cells were generated, one co-stained with PKH26 (Sigma-Aldrich, St. Louis, MO) and 500 nM of CFSE (Molecular Probes, Inc. USA) and the other stained with 5 μM or 500nM CFSE. The latter population of target cells (cells labelled with CFSE only) were then incubated for 1h with ESAT-6 peptide pool 1 (10 μg/mL—CFSE 500nM). After washing the peptide, both target cell types were adjusted and mixed to 1.5 x 10^6^ cells for each population (1:1 ratio) in 500 μL of sterile PBS. Adjusted target cells were i.v. transferred into groups of uninfected and infected mice. After 12h of target cell transfer, spleen, lungs, mediastinal and inguinal lymph nodes cell suspensions were obtained. Percentage for each target cell subpopulations was measured by flow cytometry, and killing rate for peptide-pulsed target populations was calculated according to the formula[45]:

$$\text{Killing rate}=100-\left[\left(\frac{\frac{\%\text{ Ag pulsed cells in infected mouse}}{\%\text{ Unpulsed cells in infected mouse}}}{\frac{\%\text{ Ag pulsed cells in uninfected mouse}}{\%\text{ Unpulsed cells in uninfected mouse}}}\right)\times 100\right]$$

### Statistical analysis

Groups consisted of 3 to 5 mice per time point. Results are expressed as the mean ± SE. Differences between groups were analysed using one-way ANOVA test with Tuckey’s post-test using GraphPad Prism 5.0. In the figures, *p* values of ≤ 0.05 are labeled with a single asterisk (*) in contrast to *p* values of ≤0.01 (**) or ≤0.001 (***).

## Results

### Prime-boost immunization with α-DEC-ESAT mAb induces CD4^+^ IFN-γ-producing T cells against ESAT-6 p1-peptides in uninfected mice

To determine an immunization protocol that induced ESAT-6-specific IFN-γ^+^ T cells we performed subcutaneous immunization in the footpads of naive uninfected mice with α-DEC-ESAT. Four weeks after immunization, splenocytes were stimulated for 6h with ESAT-6 peptide pools (BFA was added after 2h of stimulation) and intracellular IFN-γ staining was performed for flow cytometry analysis. With this immunization protocol the observable IFN-γ production by T cells was low and no differences were seen between α-DEC-ESAT mAb treated mice and control groups (data no shown). Positive controls showed high levels of IFN-γ in both CD4^+^ and CD8^+^ T cells (S1A Fig) from lungs, spleen, mediastinal lymph nodes (MedLN) and inguinal lymph nodes (IngLN).

We hypothesized that induction of specific T cell clones required more than one dose of α-DEC-ESAT mAb, therefore we did a prime-boost immunization protocol. After four weeks of subcutaneous priming, intranasal boost immunization was given using the same dose of antigen and adjuvant as for the priming immunization. One week later we measured the ESAT-6-specific T cell responses in the lungs, spleen, mediastinal and inguinal lymph nodes. Compared to control groups, in mice treated with a-DEC-ESAT the percentage of IFN-γ^+^ CD4^+^ T cells after stimulation with ESAT-6 p1 peptides was significantly increased in the lungs (p<0.001), MedLN (p<0.001), IngLN (p<0.001) and spleen (p<0.05) cell suspensions (Fig 1C and 1D). Very low levels of IFN-γ production were observed in p1-ESAT6 stimulated CD8^+^ T cells. Of note, the percentage of IFN-γ^+^ cells for each T cell subset (CD8^+^, CD4^+^) observed after stimulation with p2- (0.01%, 0.07%) or p3- (0.01%, 0.04%) ESAT-6 peptides (S1B Fig) showed no differences when compared to unstimulated cells (0.03%, 0.08%). We conclude that two doses of α-DEC-ESAT are required to induce specific T cell clones against ESAT-6 p1-peptides.

### ESAT-6 targeting to DEC-205^+^ APCs counteracts the delayed appearance of specific T cell responses during experimental airways tuberculosis infection

**Fig 2:**
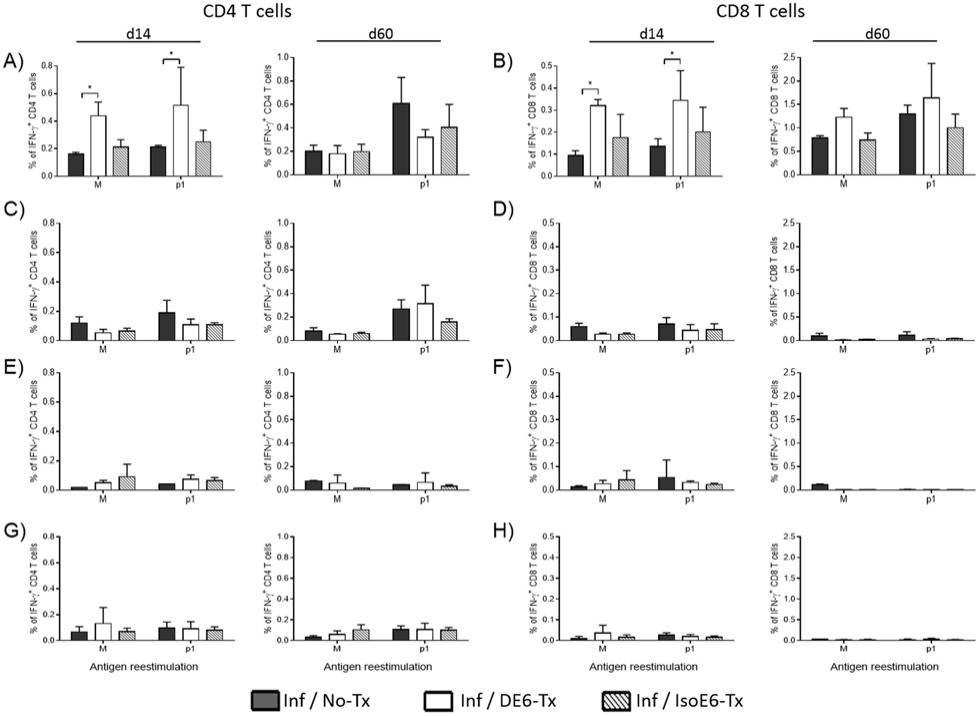
α-DEC-ESAT treatment increases IFN-γ^+^ T cells in the lung during the acute phase of experimental tuberculosis. The production of IFN-γ by CD4^+^ and CD8^+^ T cells was assessed in different tissues at two time-points during the experimental Mtb infection. α-DEC-ESAT treatment increased the percentages of IFN-γ^+^ CD4^+^ (A) and CD8^+^ (B) T cells in the lungs, during the acute (day 14) but not during the chronic (day 60) phase of the disease. In the lymphoid organs analyzed such as mediastinal lymph nodes (MedLN; C-D), inguinal lymph nodes (IngLN; E-F), and spleen (G-H), we observed very low levels of IFN-γ production either by CD4^+^ (left side panels) or CD8^+^ (right side panels) T cells. Individual mice were analyzed and 3–5 mice were used per group. Data are presented as mean plus standard error. (*) indicates P < 0.05. All bars represent groups of infected mice with different treatments. Black bars: untreated mice (Inf/No-Tx); white bars: α-DEC-ESAT-treated mice (Inf/DE6-Tx); stripped bars: mice treated with isotype control antibody conjugated with ESAT-6 (Inf/IsoE6-Tx). Cell suspensions were cultured either with medium alone (M) or medium with ESAT-6 pool 1 of peptides (p1) as detailed in Materials and Methods. MedLN = Mediastinal lymph nodes, IngLN = Inguinal lymph nodes.

To assess the effect of α-DEC-ESAT treatment in Mtb airways infection, we infected mice with Mtb H37Rv bacilli two weeks after boost immunization. At day 14 after infection, the percentage of IFN-γ^+^ CD4^+^ T cells is higher in the lungs of mice treated with α-DEC-ESAT than in non α-DEC-ESAT-treated mice (Fig 2A). As expected, α-DEC-ESAT treatment induced IFN-γ production against the immunodominant ESAT-6 epitope (p1 peptides) (Fig 2A, d14 p1). At the chronic phase of infection (d60) we observed no differences in lung IFN-γ^+^ CD4^+^ T cells as compared to controls (Fig 2A, d60). In the case of lung CD8^+^ T cells at 14 days of infection, unstimulated CD8^+^ cells showed elevated IFN-γ production, however, CD8 cells stimulated with p1 peptides showed robust IFN-γ production (Fig 2B, d14). Unlike lung CD4^+^ T cells, the production of IFN-γ by CD8^+^ T cells lasted through the chronic phase of infection (Fig 2B, d60), although the differences between unstimulated cells and p1-stimulated cells are not statistically significant the trend shows an increased percentage of IFN-γ^+^ CD8^+^ T cells in α-DEC-ESAT-treated mice. The production of IFN-γ by CD4^+^ and CD8^+^ T cells in the secondary lymphoid organs analyzed was very low and no differences were observed amongst groups of stimulated cells (Fig 2C–2H).

### Increased *in vivo* CTL killing against ESAT-6 p1-loaded target cells in the lungs of α-DEC-ESAT mAb treated mice

**Fig 3:**
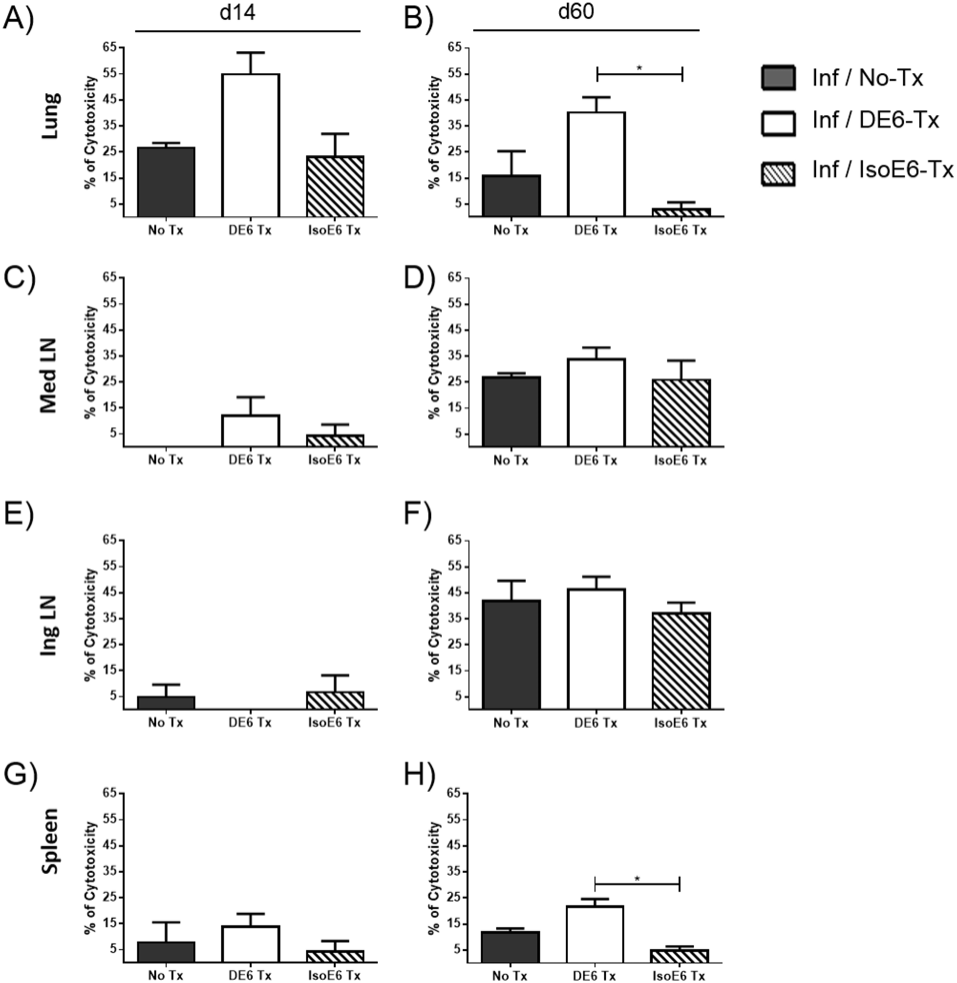
Lung *in vivo* target cell killing (CTL) rate in α-DEC-ESAT-treated mice is increased against ESAT p1 pool-loaded target cells. The CTL activity was assessed *in vivo* at day 14 (left side panels: A, C, E, G) and day 60 (right side panels: B, D, F, H) after infection with virulent Mtb H37Rv. Two types of target cells were generated and stained differentially with CFSE alone or with CFSE plus PKH26. The target cells labeled with CFSE only were loaded with ESAT-6 pool 1 (p1) of peptides. Prior to transfer into different groups of Mtb-infected mice, both subsets of target cells were combined in equal proportions. The organs evaluated were the spleen, lungs, mediastinal and inguinal lymph nodes from mice treated with α-DEC-ESAT fusion antibody (Inf/DE6-Tx, white bars); and mice which received the isotype control antibody conjugated with ESAT-6 (Inf/IsoE6-Tx, stripped bars). Individual mice were analyzed and 3–5 mice were used per experimental group. Data are presented as mean plus standard error and percentage of cytotoxicity was calculated as indicated in Methods. (*) indicates P<0.05. All bars represent infected mice with different treatments for each group, as indicated.

To assess the effect of ESAT-6 targeting to DEC205^+^ APC on cytotoxic T cell responses, we performed the *in vivo* CTL killing assay in the lungs, spleen, MedLN, and IngLN, against target cells loaded *in vitro* with ESAT-6 p1 peptides. Our results showed that in the lungs of α-DEC-ESAT treated mice, the killing rate for p1-loaded target cells at day 14 post-infection was doubled compared to the killing rate observed in control groups (Fig 3A). Sixty days after infection the killing rate of target cells in the lungs decreased, however, this rate remained significantly higher than in Iso-ESAT—treated mice (Fig 3B). In the lymphoid organs assessed (mediastinal and inguinal lymph nodes, and spleen), we found no differences in *in vivo* CTL killing amongst the different treatment groups (Fig 3C–3H), with one exception, in the spleen at day 60 the killing rate for p1-loaded target cells was significantly higher in α-DEC-ESAT treated group when compared to the Iso-ESAT—treated group (Fig 3H).

### Bacterial burden and cellular infiltrate in the lungs of α-DEC-ESAT-treated mice

**Fig 4:**
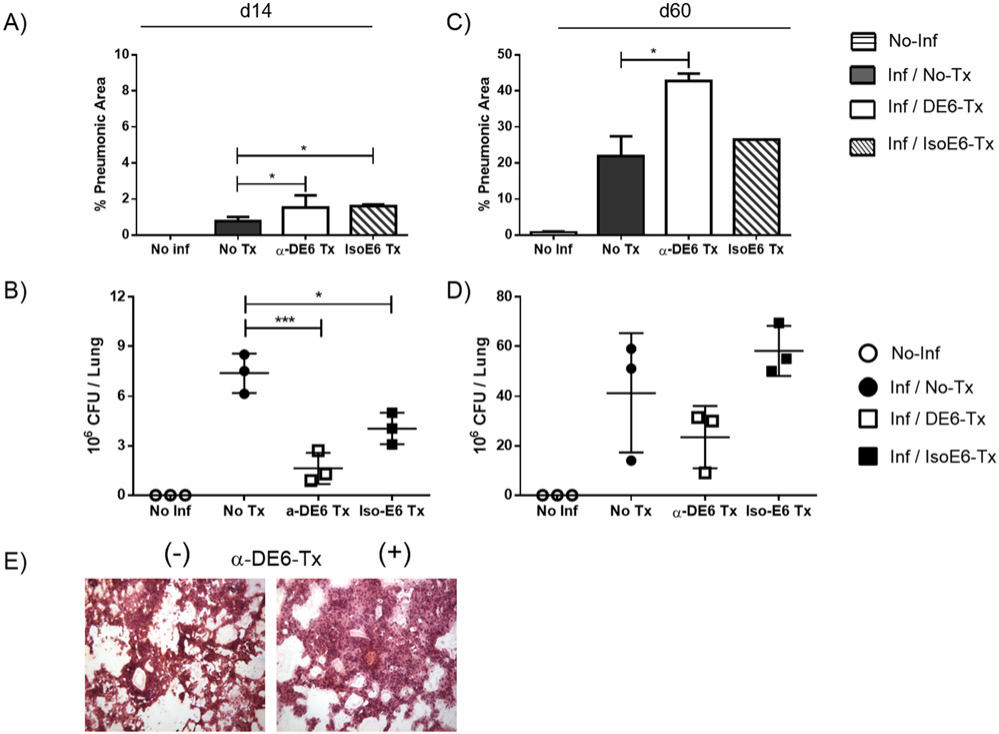
α-DEC-ESAT-treated mice show reduced bacterial burden and increased cellular infiltrate in the lungs. Pulmonary mycobacterial load (CFUs/lung) and cellular infiltrate (% pneumonic areas) were quantified at two time points during Mtb infection in the different experimental groups. Horizontally stripped bars indicate uninfected non-immunized mice (No-Inf). All other bars represent infected mice with different treatments. Black bars: untreated mice (Inf/No Tx); white bars: α-DEC-ESAT-treated mice (Inf/DE6-Tx); diagonally stripped bars: mice treated with isotype control antibody-ESAT (Inf/IsoE6-Tx). Quantification of the cellular infiltrate during the acute infection is shown in (A) while that of the chronic stage is shown in (C). The bacterial burden expressed as CFUs per lung is shown in (B) for the acute stage of the infection and in (D) for the chronic phase. (E) Representative picture of HE staining of lungs from infected mice which were α-DEC-ESAT-untreated (-) or α-DEC-ESAT-treated (+). Individual lungs were analyzed by duplicate and 3 mice were used per group. Data are presented as mean plus standard error. (*) Represents P<0.05.

To test the possibility of *in situ* immune protection induced by α-DEC-ESAT treatment we measured both the cell infiltrate and the bacterial burden in the lungs. During the acute phase (day 14) of infection, very few pneumonic areas, mostly perivascular, were found in the lungs of all groups tested (Fig 4A). However, the Mtb load in the lungs of untreated mice was 3-fold higher than that of α-DEC-ESAT treated animals (Fig 4B).

At the chronic phase of infection, α-DEC-ESAT-immunized mice showed more infiltrate than untreated or than isotype control mAb-treated mice (Fig 4C); however, tissue damage was more evident in the lungs of untreated animals.

Of note, at day 60 the lungs of mice that were immunized with α-DEC-ESAT showed lower mycobacterial burden (~50% less) than mice infected but not treated with α-DEC-ESAT (Fig 4D). Our histological evaluation revealed a prominent cellular infiltrate (Fig 4E) in α-DEC-ESAT treated group. In light of our other findings, elevated IFN-γ production and CTL activity, we suggest these results might be the consequence of a robust cellular immune response within the mycobacterial target organ.

## Discussion

Tuberculosis is a re-emerging global health problem that yearly causes around 1.7 million deaths. Theoretically, Mtb success as a human pathogen might rely on evasion mechanisms and on counteracting immune responses initiated early upon the natural process of infection. For instance, immune evasion mechanisms already shown for mycobacteria include interruption of phagosome/lysosome maturation[12,13], downregulation of proinflammatory cytokines[1,39], inhibition of cytotoxicity[17], and more recently described, an active delay of the induction of specific T cell responses [28,46]. The lack of a more efficacious vaccine has led to increased efforts to understand both the host-pathogen interactions *in vivo* and the pathogenesis of tuberculosis.

Recent works indicate that Mtb airways infection delays the onset of T cell activation in the mediastinal lymph nodes. In fact, the peak influx of DCs to the regional lymph nodes and of IFN-γ producing T cells to the lungs occurs around the third week post-Mtb infection [20,47]. This contrasts with other, similar infectious models where DCs accumulate much earlier, in about 24–48 h [48,49]. Inducing T cell IFN-γ production before 3 weeks post-infection might limit mycobacterial growth in the lungs; for example, by i.v. inoculation of Mtb[50] or by transferring ESAT-6-specific tg-TCR T cells[5,50].

Dendritic cells are widely distributed in mucosal and epithelial surfaces and can be mobilized to efficiently present antigens and prime naïve T cells, therefore DCs are good candidates to target microbial antigens for prophylaxis. Using monoclonal antibodies to target antigens to DCs might be a new way to enhance and perhaps to accelerate protective T cell responses and circumvent the natural, adaptive evasion mechanisms developed by pathogens. Targeting Ag to DCs has demonstrated that, compared to standard procedures, very low Ag doses are required to induce vigorous, Ag-specific T cell responses [33,51,52]. Besides, by direct delivery to DCs, Ag targeting limits unnecessary Ag diffusion and perhaps more important, excessive Ag degradation by macrophages [53,54]. Consequently, this increases the absolute amount of Ag available in the DC population, likely making Ag presentation for infrequent Ag-specific T cells much more efficient. Furthermore, direct Ag targeting to certain endocytic receptors expressed by DCs (e.g. DEC205) might circumvent the risk of potentially evasive mechanisms of pathogens during the natural process of infection.

In our study, we targeted ESAT-6, a mycobacterial antigen, to DEC205^+^ APCs and assessed the effects over protection-associated T cell responses, namely IFN-γ production and CTL activity. In the mouse lungs, DEC205 is expressed by macrophages and a subset of epithelia-associated DCs; in our experimental design, giving α-DEC-ESAT-6 intranasally would target both DCs and macrophages, however only the former is capable to migrate and prime T cells in the draining lymph node [55]. ESAT-6 is a secreted mycobacterial protein present only in pathogenic mycobacteria thus absent in BCG[56] (the vaccine most widely used). Besides its association with virulence, ESAT-6 induces strong Th1-type T cell responses and *in vivo* protection in certain models [42,43]. Our results showed that targeting ESAT-6 to DEC205^+^ APCs induced IFN-γ production in T cells and an increase in *in vivo* target cell killing, thus we surmise that ESAT-6 targeting ultimately leads to efficient Ag presentation by DEC205^+^ APCs.

In uninfected mice that received two doses of α-DEC-ESAT, ESAT-p1-specific IFN-γ^+^ T cells were readily induced in all the organs tested. Considering the potential size of the TCR repertoire[57], the IFN-γ response obtained from polyclonal stimulation, the approximate quantity of ESAT-6 delivered in 5μg of α-DEC-ESAT (~370ng), and the percentage of IFN-γ^+^ specific-T cells obtained after ESAT-6 targeting to DEC205^+^ APCs, suggests that α-DEC-ESAT treatment is an effective strategy to induce activation of infrequent Ag-specific CD4^+^ T cells. Indeed, these results agree with previous reports showing that either OVA or the mycobacterial Ag85 targeting to DEC205^+^ cells, readily promotes the proliferation of specific T cells [35,51,58].

In α-DEC-ESAT-treated animals, we observed that at 14 days post-infection, and in contrast with results obtained in α-DEC-ESAT-untreated mice, the lungs had a high percentage of ESAT-6-specific IFN-γ-producing T cells, elevated *in vivo* target cell killing, and a considerable reduction of lung bacterial burden.

We hypothesize that, in α-DEC-ESAT-treated mice, a relatively high percentage of activated ESAT-6-specific memory T cells might be available before infection, during the span between boost immunization and the infection. During the first three weeks after infection, Mtb secretes ESAT-6 into the lungs, permitting effector p1-specific T cells to be rapidly recruited to the lungs[59] of α-DEC-ESAT-treated mice, a process that would only occur until 21 days post-infection in untreated mice. The increase of IFN-γ production in the lungs might favor a local proinflammatory microenvironment, as well as migration and maturation of Mtb-Ag-loaded DC to regional (mediastinal) lymph nodes where activation of broader Mtb-specific IFN-γ-producing T cell responses may occur.

In untreated mice, our model of pulmonary tuberculosis at the chronic infection stage (d60) is characterized by elevated lung CFUs and by increased production of IL-4 while IFN-γ production remains elevated[60,61]. In our experiments, at day 60 post-infection the CD8^+^ T cell responses (IFN-γ production and *in vivo* CTL killing) in the α-DEC-ESAT-treated mice remained increased, likely contributing to mycobacterial control. In agreement with previous reports[35], only CD8^+^ T cells from α-DEC-ESAT treated mice showed high levels of IFN-γ in the lungs, likely because targeting antigens to DEC205 receptor favors a more prolonged MHC-I-antigen presentation *in vivo*.

*In vivo* CTL killing rate has been barely assessed in experimental pulmonary tuberculosis. In the *in vivo* CTL killing assays, CTL activity is indirectly measured by the disappearing of target cells loaded with specific microbial antigens relative to target cells that contain no microbial antigens. During infection, killing of Mtb infected cells leads to bacterial death or to uptake of bacilli by activated macrophages[62]. While CD4^+^ T cells limit Mtb infection during the acute phase of infection, cytotoxic and IFN-γ producing CD8^+^ T cells seem the main protective subpopulation during the chronic phase[63]. Antigen targeting to DEC205^+^ cells induces a more prolonged antigen presentation via MHC-I[35], thus promoting specific-CD8^+^ T cell activation and, probably, increased *in vivo* CTL killing. Indeed, in our study, a greater rate of *in vivo* p1-loaded target cell elimination is seen in the lungs of mice treated with α-DEC-ESAT than in (α-DEC-ESAT) untreated mice. Presumably the cytolysis observed is mediated by ESAT-6-specific T cells generated after α-DEC-ESAT immunization. At day 60 post-infection, *in vivo* target cell killing in the spleen appears increased, this was expected at this time point since the infection is disseminated and a systemic immune response is generated trying to contain the infection.

Finally, the efficacy of this approach, antigen targeting *in vivo* using monoclonal antibodies to DCs, has been barely studied in experimental tuberculosis. While preparing this manuscript, one report showed that targeting the mycobacterial Ag85 to DEC205^+^ APCs increased IFN-γ production but showed limited effect on the bacterial load. However, neither lung histopathology nor *in vivo* CTL killing assays were assessed in the study mentioned above. Our results revealed that antigen selection can have important, differential effects upon the outcome of this strategy. ESAT-6 targeting to DEC205^+^ APCs prior to infection importantly reduces the onset time for specific-CD4^+^ and CD8^+^ T cell responses, correlating with cellular infiltrate, *in vivo* CTL killing assays and, ultimately, with reduced bacterial load in the lungs. Even though we immunized with rather small amounts of ESAT-6, an antigen associated with mycobacterial virulence and with restricted recognition by T cells, targeting to DEC205^+^ APCs induced T cell responses and a significant reduction in bacterial burden *in situ* in the Mtb target organ.

## Supporting Information

S1 Fig IFN-γ production by cell suspensions stimulated with α-CD3 antibody, ESAT-6 p2 or p3 peptide pools in different tissues of non-infected mice. Lung, spleen, mediastinal and inguinal lymph nodes cell suspensions were stimulated *ex-vivo*. Representative dot plots of IFN-γ production under (A) polyclonal stimulation with α-CD3 antibody or (B) peptide pool p2 or peptide pool p3 of ESAT-6 peptide library. No IFN-γ is induced with either p2 or p3 peptide pools.
(TIF)
