# Supplementary figures and images for: ESAT-6 Targeting to DEC205+ Antigen Presenting Cells Induces Specific-T Cell Responses against ESAT-6 and Reduces Pulmonary Infection with Virulent Mycobacterium tuberculosis
Source: PLoS One. 2015 Apr 27;10(4):e0124828. doi: 10.1371/journal.pone.0124828 (PMC4411092; doi:10.1371/journal.pone.0124828)

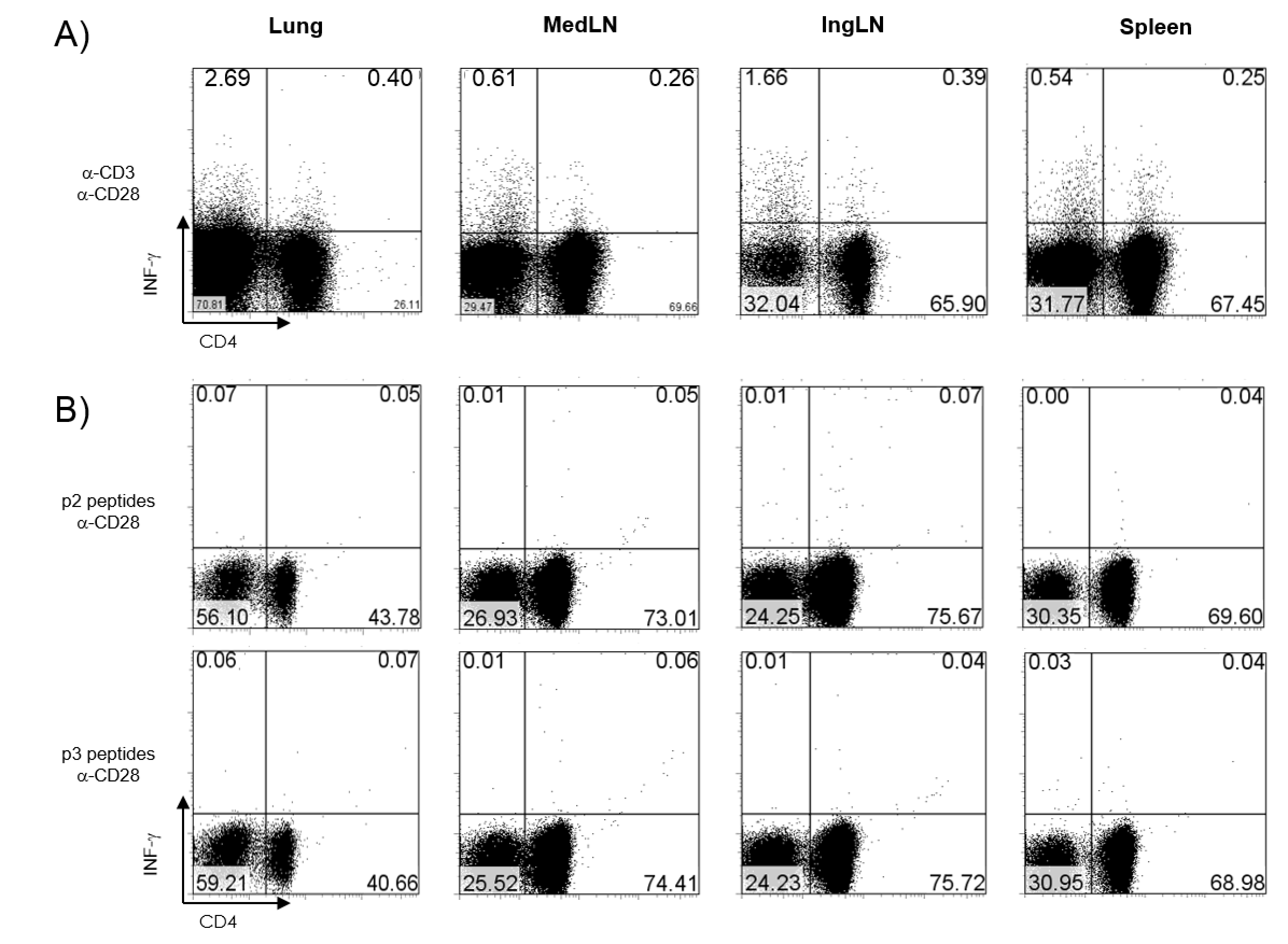

Supplement: S1 Fig — Lung, spleen, mediastinal and inguinal lymph nodes cell suspensions were stimulated ex-vivo. Representative dot plots of IFN-γ production under (A) polyclonal stimulation with α-CD3 antibody or (B) peptide pool p2 or peptide pool p3 of ESAT-6 peptide library. No IFN-γ is induced with either p2 or p3 peptide pools. (TIF) [file pone.0124828.s001.tif]
